# Meta-analysis of the relationship between bullying and depressive symptoms in children and adolescents

**DOI:** 10.1186/s12888-023-04681-4

**Published:** 2023-03-30

**Authors:** Zixiang Ye, Dongmei Wu, Xiaoyan He, Qin Ma, Jianyan Peng, Guoju Mao, Lanling Feng, Yuhao Tong

**Affiliations:** 1 Nursing Department, The Clinical Hospital of Chengdu Brain Science Institute, MOE Key Laboratory for Neuroinformation, University of Electronic Science and Technology of China, Chengdu, China; 2 Nursing Key Laboratory of Sichuan Province, Chengdu, China; 3 Affiliated Hospital of Chengdu University, Chengdu, China

**Keywords:** Bullying, Depression, Children, Adolescents, Meta-analysis

## Abstract

Childhood and adolescence are critical periods for physical and mental development; thus, they are high-risk periods for the occurrence of mental disorders. The purpose of this study was to systematically evaluate the association between bullying and depressive symptoms in children and adolescents. We searched the PubMed, MEDLINE and other databases to identify studies related to bullying behavior and depressive symptoms in children and adolescents. A total of 31 studies were included, with a total sample size of 133,688 people. The results of the meta-analysis showed that the risk of depression in children and adolescents who were bullied was 2.77 times higher than that of those who were not bullied; the risk of depression in bullying individuals was 1.73 times higher than that in nonbullying individuals; and the risk of depression in individuals who bullied and experienced bullying was 3.19 times higher than that in nonbullying-bullied individuals. This study confirmed that depression in children and adolescents was significantly associated with being bullied, bullying, and bullying-bullied behavior. However, these findings are limited by the quantity and quality of the included studies and need to be confirmed by future studies.

## Background

Depression refers to a persistent change in mood such as feelings of loss, sadness, and hopelessness [1]. According to the World Health Organization (WHO) report, the number of depressed people worldwide has reached 264 million [2]. Epidemiological studies and clinical interviews show that children and adolescents have high incidence rates of depression, ranging from 2 to 8%. The detection rate of depression in adolescents in China is 15.7—29% [3]. Although depression among adolescents can disappear, 40—70% of children and adolescents still have the possibility of relapse within 5 years [4]. Indeed, depression is one of the most common mental health problems in children and adolescents and mainly manifests as a persistent decline in academic performance, feelings of worthlessness, difficulty in making friends, and poor sleep quality [5, 6]. Depression is very common among adolescents and affects adolescents’ interpersonal relationships, academic performance, and hobbies as well as their physical and mental health; severe depression may even be life-threatening [7]. Suicide is the third leading cause of death among children and adolescents, and depression is the leading cause of suicide among adolescents. A study in China reported that the incidence of nonsuicidal self-injury (NSSI) in children and adolescents with depression was as high as 44.0 to 61.2% [8].

In recent years, given rapid advances in news coverage and social media, harmful incidents in schools have been reported, and school bullying of children and adolescents has attracted more attention from society as a whole. Children and adolescents are exposed to the social environment during their growth. In this environment, bullying is used to solve conflicts. They are more likely to show aggressive behavior and regard bullying as a way to solve conflicts [9]. Between 2005 and 2013, the incidence of bullying in American schools was between 20 and 30% [10]. In Chinese middle school students, the bullying rate is 1.68% ~ 10.60%, the victimization rate is 5.91% ~ 25.70%, and the bullying victimization rate is 3.28% ~ 14.70% [11]. Additionally, with the advent of the internet era, cyberbullying has emerged as a new form of bullying; the incidence of cyberbullying has increased each year, attracting attention from researchers worldwide. A study by Pillkey and Jacqueline found that 37.8% of students experienced cyberbullying, 56% of students had witnessed cyberbullying, and the incidence of cyberbullying among eighth-grade students was as high as 42.1% [12]. Studies by Sampasakanyinga [13] and Hinduja [14] reported that victims of cyberbullying and school bullying have significantly greater suicide intent. Compared with people who were not bullied, the incidence of negative outcomes such as depression, anxiety, suicide, and loneliness was higher among those who were bullied. In addition, because the bully has no experience of being bullied, it is easy to ignore the harm caused by the bullying behavior, while people who are bullying and being bullied are more able to perceive the pain caused by the bullying; therefore, the twin pressures of bullying and being bullied combined with poor social and psychological function can increase the likelihood of depression, anxiety and other negative feelings [15, 16]. It can be seen that bullying, being bullied and bullying-being bullied should all receive higher social attention, and the physical and mental development of children and adolescents should not be ignored.

In recent years, studies in China and other countries have found that bullying and being bullied predict the occurrence of depression in children and adolescents [17]. In general, in the past meta-analysis, bullying and bullying—being bullied were not included in the study, and the relationship between the above three factors and depression was unclear. Therefore, the aim of the present study was to summarize the relevant research and explore associations through a meta-analysis. Specifically, the investigated associations included the relationships of bullying, being bullied, bullying and being bullied (hereafter, bullying-bullied) with depressive symptoms in children and adolescents. Ideally, these findings will inform and guide the development of preventive measures, thereby promoting the physical and mental health development of children and adolescents.

## Methods

### Protocols and registration

The study protocol was prospectively registered on the International Platform of Registered Systematic Review and Meta-analysis Protocols (INPLASY) database (registration number: 202270087). The study was conducted based on the Cochrane Collaboration’s guidelines. The screening of eligible studies and data reports was conducted based on the Preferred Reporting Items for Systematic Reviews and Meta-Analyses (PRISMA) statement [18].

### Literature search strategy

The PubMed, MEDLINE, Embase, Cochrane Library, CNKI, and WanFang databases were electronically searched to identify relevant studies on bullying and depression in children and adolescents. The Chinese search terms included “children”, “adolescents”, “bullying”, “bullied”, “depression”, etc. The English search terms included “adolescent”, “teen”, “teenager”, “youth”, “female”, “male”, “child”, “children”, “bullying”, “depression”, “depressive symptoms”, “emotional depression”, etc. The search was carried out using a combination of subject headings and keywords.

### Eligibility criteria

#### Inclusion criteria

We included cross-sectional studies on the association between bullying and depression in children and adolescents. Eligible studies were published in Chinese or English, and the main subjects were children and adolescents, ranging in age from 6 to 18 years old. Bullying behavior included verbal bullying and was defined as follows: in the past 12 months, an individual or group engaged in persistent, repeated negative behavior toward other individuals or groups, such as verbal behavior (e.g., ridicule, nicknames, or spreading rumors to isolate others) or physical contact (e.g., hitting, kicking, or shoving). Being bullied was defined as an individual or group subjected to the above behavior by other individuals or groups. Finally, bullying-bullied refers to individuals that both bullied and were bullied by other individuals. The outcome measure was the incidence of depression, based on clear diagnostic criteria in the literature [19].

#### Exclusion criteria

The exclusion criteria were as follows: (1) studies not published in Chinese or English, (2) duplicate studies, (3) studies with unavailable data, and (4) studies lacking important information that was unable to be obtained (i.e., the author was contacted but did not respond).

### Literature screening and data extraction

Two reviewers independently screened the literature, extracted the data and cross-checked it. In cases of disagreement, a third party was consulted to reach a decision. We also contacted the authors of studies to obtain important information not reported in the publication. During the literature search, potentially relevant studies were identified by screening the title and abstract; after excluding obviously irrelevant studies, the full text of these studies was reviewed to determine its eligibility for inclusion. The data extracted included the following: (1) basic information regarding the included studies, including the first author and publication date; (2) baseline characteristics of the research subjects, including the sample size of each group and the age and sex of the participants; (3) the specific methodology (e.g., follow-up duration); (4) key elements related to the risk of bias assessment; and (5) relevant outcome indicators and outcome assessments.

### Risk of bias in the included studies

Cross-sectional studies were assessed for risk of bias using the quality assessment criteria recommended by the Agency for Healthcare Research and Quality (AHRQ) [20]. This scale has 11 items, each of which are scored as “Yes” (1 point) or "No/Unclear" (0 points), for a total of 11 potential points. A score of 0–3 indicates low-quality literature, 4–7 indicates medium-quality literature, and 8–11 indicates high-quality literature.

### Statistical analysis

Stata 14.0 was used for statistical analysis, and the odds ratio (OR) and 95% confidence interval (CI) are reported to indicate effects. The χ^2^ test (test level α = 0.1) and *I2* statistic were used to determine the size of heterogeneity among the results of the included studies. When *P* ≥ 0.1 and *I*^*2*^ < 50%, the heterogeneity of the included literature was low, and a fixed-effect model was used for the meta-analysis. In contrast, *P* < 0.1 and *I*^*2*^ ≥ 50% indicated that the included studies had nonnegligible heterogeneity, and a random-effects model was used for meta-analysis. Sensitivity analysis or subgroup analysis was performed on the results of studies with large heterogeneity, and studies were excluded if necessary to ensure the reliability and stability of the study results. Egger's test was used to assess publication bias.

## Results

### Literature search

**Fig. 1:**
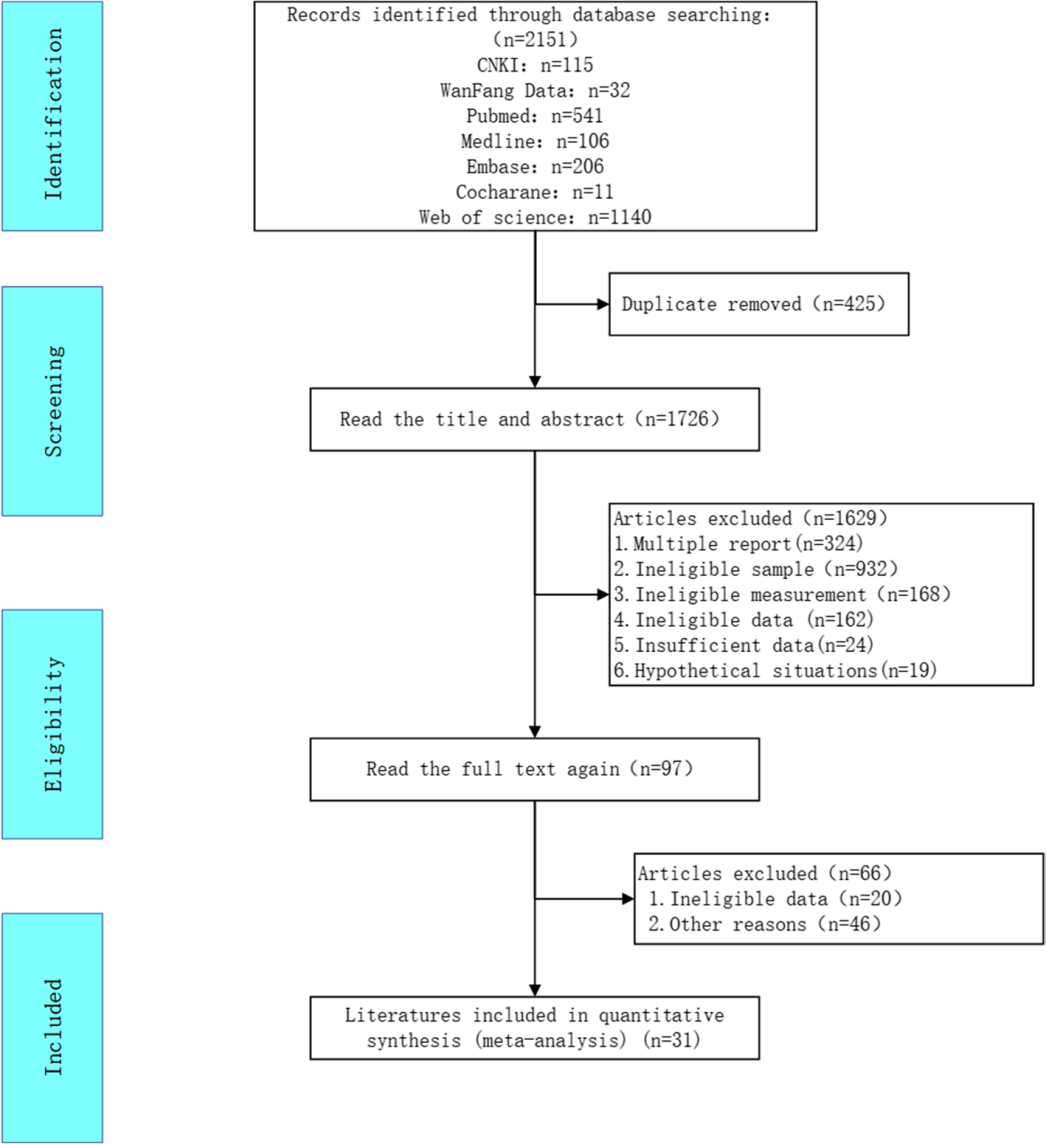
PRISMA flow chart of the study selection process

Figure 1 presents the PRISMA [18] flow chart of the study selection and exclusion process. An electronic database search identified 1,989 records. After sorting and eliminating duplicates, 425 articles were screened by title and abstract. A total of 97 relevant full-text articles were assessed for eligibility, of which 31 were cross-sectional surveys and included in the final analysis. The most common reason for exclusion was a lack of available data.

### Quality assessment

Table 1 shows the characteristics of all included studies. A total of 31 studies were included in this meta-analysis. The overall quality score of the articles had an average of 6.94 ± 1.00 points; this indicates a moderately high quality, ranging from high-quality literature (a maximum of 8 points) to medium-quality literature (a minimum of 5 points). Most articles did not describe how they evaluated and/or controlled for confounders and did not explain how missing data were handled in the analysis.

**Table 1:** Description of studies included in the systematic review and meta-analysis

Study (first author, publication year)	Country/continent	Sampling procedures	Age or grade range (M; SD)	N; female (%)	Research categories	Depression measurement	Bias risk assessment	Bullying type
Chen JY, 2022 [21]	China/Asia	Cluster random sampling (school)	9–18 (12.85; 1.70)	2931 (48.24)	①②③	CES-D-10	8	Cyber
Wang WK, 2021 [22]	China/Asia	Cluster random sampling (school)	7th-11th (14.6; 1.60)	211 (43.96)	①②③	CES-D	7	Traditional
Xie Y, 2021 [23]	China/Asia	Cluster random sampling (school)	12–18 (14.85; 1.27)	3531 (49.50)	①②③	SDS	8	Traditional
Liu XQ, 2021 [24]	China/Asia	Cluster random sampling (school)	junior and high school students (14.8; 1.6)	8252 (52.00)	①②③	PHQ-9	8	Traditional
Cheng T, 2020 [25]	China/Asia	Cluster random sampling (school)	high school students (N/S)	5974 (48.18)	①	CES-D	8	Traditional
Zhang ZW, 2019 [26]	China/Asia	Cluster random sampling (school)	6th-8th (N/S)	1649 (49.55)	①②③	PHQ-9	7	Traditional
Kim JH, 2016 [27]	Korea/Asia	Cluster random sampling (school)	9—18 (N/S)	3627 (52.69)	①	Single item	7	Traditional
Abd Razak MA, 2019 [28]	Malaysia/Asia	Cluster random sampling (school)	teenagers	27,399 (50.40)	①	DASS-21	8	Traditional
Rothon C, 2011 [29]	England/Europe	Simple random sampling (school)	11—14 (N/S)	2734 (51.40)	①	SMFQ	6	Traditional
Alrajeh SM, 2021 [30]	Qatar/Asia	Simple random sampling (school)	16–18 (N/S)	836 (78.00)	①②③	PHQ-9	8	Traditional
Donato F, 2021 [31]	Italy/Europe	N/A (school)	15–16 (15.2; 0.40)	3002 (55.90)	①	CES-DC	6	Traditional
Jung YE, 2014 [32]	Korea/Asia	Simple random sampling (school)	11–14 (N/S)	4513 (48.90)	①②③	Single item	7	Cyber
Lemstra ME, 2012 [33]	Canada/North America	**General survey sampling (school)**	10–16 (N/S)	4197 (49.00)	①	CES-D 12	8	Traditional
Selkie EM, 2014 [34]	USA/North America	General surveysampling (school)	18–25 (N/S)	265 (100)	①②③	PHQ-9	5	Cyber
Kaur J, 2014 [35]	Malaysia/Asia	Cluster random sampling (school)	13–17 (N/S)	28,738 (49.80)	①	Single item	7	Traditional
Hansson E, 2019 [36]	Iceland/Europe	Cluster random sampling (school)	11–15 (13; 1.61)	11,018 (47.30)	①	Single item	8	Traditional
Schneider SK, 2011 [37]	USA/North America	General surveysampling (school)	9th-12th (N/S)	20,406 (49.60)	①	Single item	6	Traditional/cyber
Liu X, 2020 [38]	China/Asia	Cluster random sampling (school)	10–18 (N/S)	5926 (55.00)	①	PHQ-9	7	Traditional
Ybarra ML, 2015 [39]	USA/North America	Simple random sampling (school)	13–18 (15.8; 1.6)	5542 (51.00)	①	CESD-R	8	Cyber
Andre S, 2010 [40]	Finland/Europe	N/A (school)	13–16 (N/S)	2215 (47.89)	①②③	Single item	7	Cyber
Hemphill SA, 2015 [41]	Australia/Oceania	Cluster random sampling (school)	10th (16; 0.4)	791 (54.00)	①	SMFQ	6	Cyber
Messias E, 2014 [42]	USA/North America	Cluster random sampling (school)	9th-12th (N/S)	5,425 (50.2)	①	Single item	8	Traditional/cyber
Klomek AB, 2008 [43]	USA/North America	Cluster random sampling (school)	13–19 (14.8; 1.2)	2181 (41.9)	①	BDI-IA	6	Cyber
Goebert D, 2011 [44]	USA/North America	N/A (school)	high school students (N/S)	677 (60.2)	①	Multiple items	6	Cyber
Sampasa-Kanyinga H, 2014 [13]	Canada/North America	Simple random sampling (school)	7th-12th (14.5; 1.80)	3509 (54.9)	①	Single item	7	Traditional
Landstedt E, 2014 [45]	Sweden/Europe	General survey sampling (school)	13–16 (N/S)	1214 (52.7)	①	CES-D	5	Traditional/cyber
Chang FC, 2015 [46]	China/Asia	Cluster random sampling (school)	7th–9th (N/S)	1867 (51.7)	①②	CES-D	8	Cyber
Islam MI, 2020 [47]	Australia/Oceania	Cluster random sampling (school)	12–17 (14.83; 1.70)	2166 (47.8)	①	DISC-IV	6	Traditional/cyber
Mereish EH, 2019 [48]	USA/North America	Cluster random sampling (school)	10–18 (N/S)	1129 (51.7)	①	Single item	7	Cyber
Mallik CI, 2019 [49]	Bangladesh/Asia	Simple random sampling (school)	14–17 (15.7; 1.03)	276 (34.1)	①	DAWBA	5	Cyber
Elgar FJ, 2014 [50]	USA/North America	Simple random sampling (school)	12–18 (15; 1.7)	18,834 (50.5)	①②	N/S	7	Traditional

*N/S* not specified, *M* mean, *SD* standard deviation, *N* total number of participants, *USA* the United States of America, *SMFQ* Short Mood and Feelings Questionnaire, *DSM-IV* Diagnostic Statistical Manual of Mental Disorders, *BDI-IA* Beck Depression Inventory (version I, amended), *CES-D* Center for Epidemiologic Studies Depression Scale, *CESD-R* Center for Epidemiologic Studies Depression Scale Revised, *CESD-10* Centers for Epidemiological Studies Depression Survey-10, *DASS-21* Depression, Anxiety, and Stress Scale-21, *PHQ-9* Patient Health Questionaire-9, *SDS* Self-rating Depression Scale, *DISC-IV* Diagnostic Interview Schedule for Children Version IV, *DAWBA* Development and Well-Being Assessment

Research categories: ① Bullied, ② Bullying, ③ Bullying-bullied

### Description of included studies

In most studies, female participants made up the majority (*n* = 18) (Table 1). Most studies were conducted in Asia (*n* = 14), especially in China, while other studies were from Europe (*n* = 13) and Oceania (*n* = 2). Most studies included adolescents of multiple ethnicities (*n* = 10). All research subjects were from schools, including middle and high schools. The research categories included bullying individuals (*n* = 31), bullied individuals (*n* = 11), and bullying-bullied individuals (*n* = 9). The bullying types included traditional bullying (*n* = 16), cyberbullying (*n* = 11), and both (*n* = 4).

Most studies assessed cyberbullying with a single measure (*n* = 9). For the assessment of depression, four studies used only one item. However, this single item assessed the two main symptoms of depression, "feelings of sadness and despair for approximately two weeks," which are equivalent to dysphoria and anhedonia.

#### Depression

Two studies did not specify an assessment item for depression [44, 50]. Data reported in ten studies were raw, and only crude ORs were extracted [21–25, 28, 33, 39, 47, 49]. Only three studies assessed differences between depression outcomes and frequency of cyberbullying [31, 46, 50]. Another study assessed differences in depression outcomes among two different groups of victims: cyberbullying victims (defined as those who experienced repeated bullying and a power imbalance) and victims of "generalized" cyberharassment [39].

### Relationship between being bullied and depression in children and adolescents

**Fig. 2:**
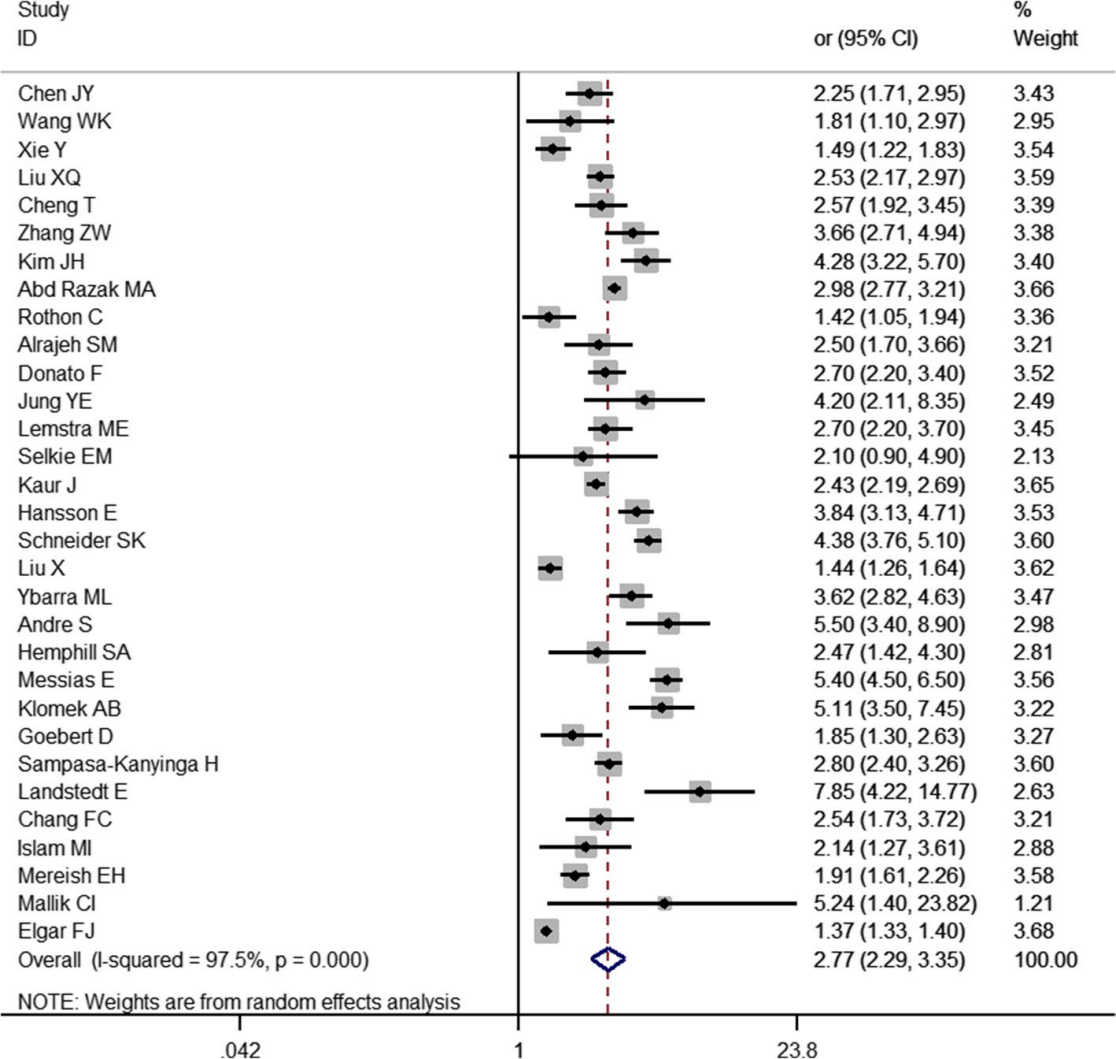
Meta-analysis of the relationship between being bullied and depression in children and adolescents

**Fig. 3:**
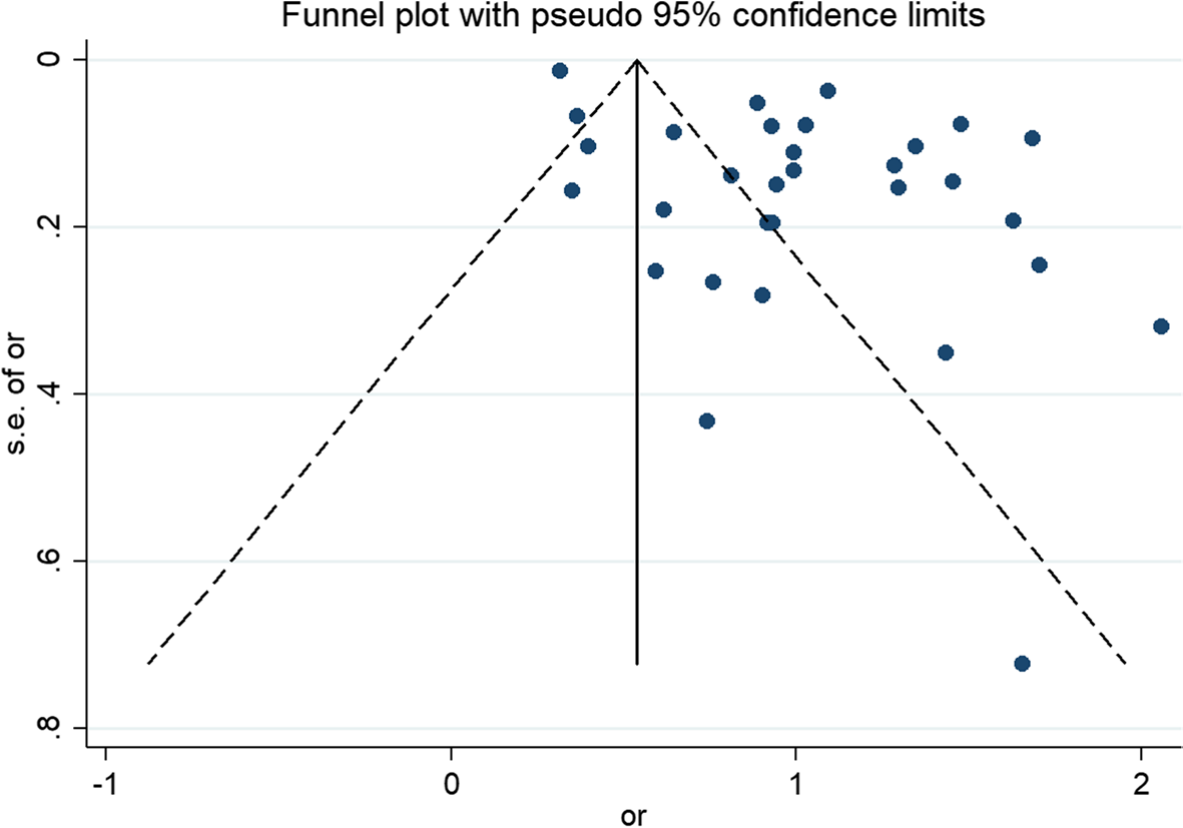
Funnel diagram of the relationship between bullying and depression in children and adolescents

A total of 31 studies were included in this analysis. The random-effects model showed that the risk of depression in children and adolescents who were bullied was 2.77 times higher than in those who were not bullied [*OR* = 2.77, 95% CI (2.29,3.35), *P* < 0.001] (Fig. 2). Sensitivity analysis was conducted by individually excluding each study; this analysis found no substantial change in the results, suggesting a stable pooled effect size (*OR* = 1.02, 95% *CI* = 0.83, 1.21). However, the funnel plot was obviously asymmetric (Fig. 3), and the results of Egger's test (*t* = 4.64, *P* < 0.05) suggest the presence of publication bias.

Taking the average age of the subjects as the covariate, the restricted maximum likelihood method was used for the single-factor meta-regression analysis. The results showed that there was a weak correlation between the age of the subjects and the occurrence of depression after being bullied – that is, age was not the source of heterogeneity in this study [B = -0.02, 95% CI (-0.33, 0.29)].

### Relationship between bullying and depression in children and adolescents

**Fig. 4:**
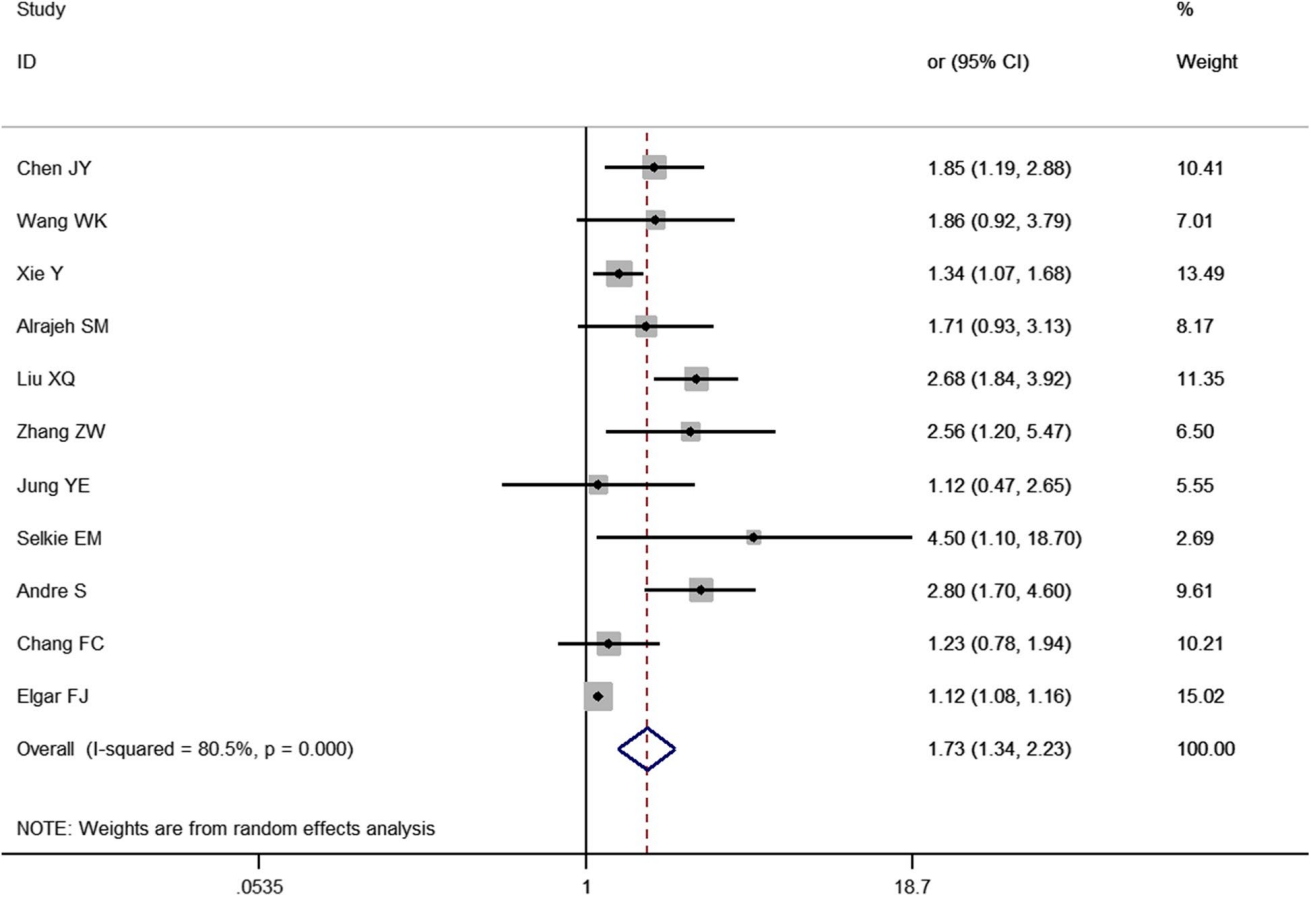
Meta-analysis of the relationship between bullying and depression in children and adolescents

A total of 11 studies were included in this analysis. The random-effects model showed that the risk of depression in bullying individuals was 1.73 times higher than that in nonbullying individuals [*OR* = 1.73, 95% *CI* (1.34, 2.23), *P* < 0.0001] (Fig. 4).

### Relationship between bullying-bullied behavior and depression in children and adolescents

**Fig. 5:**
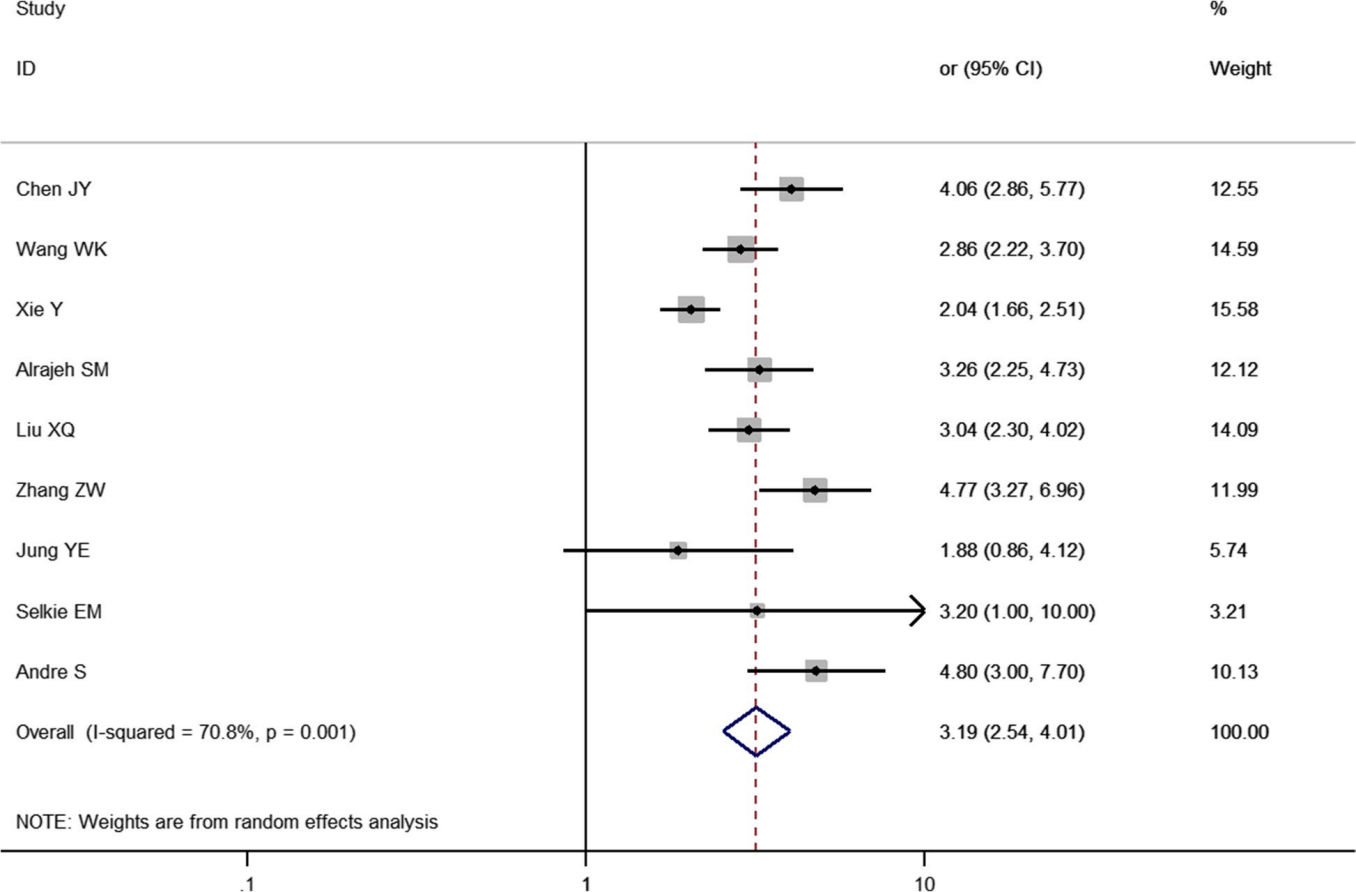
Meta-analysis of the relationship between bullying-bullied behavior and depression in children and adolescents

A total of 9 studies were included in this analysis. The results of the random-effects model showed that the risk of depression in those who both bullied and were bullied was 3.19 times higher than in nonbullying-bullied individuals [*OR* = 3.19, 95% *CI* (2.54, 4.01), *P* = 0.001] (Fig. 5).

### Subgroup analysis

Subgroup analysis was conducted based on sex, publication year, cultural background, sampling method, bullying type and article quality. The results showed significant differences in the effect size of the relationships between being bullied and depression in children and adolescents with different cultural backgrounds and bullying types (*P* < 0.001); the effect sizes were relatively low in studies from Asia and those on cyberbullying (Table 2).

**Table 2:** Results of subgroup analysis of the association between bullying and depression in children and adolescents

Group	Number of included studies	Heterogeneity		Meta-analysis results	
		*I*^*2*^(%)	***P***	*OR***(95%* CI*)**	***P***
**Sex**					
Males and females	30	97.6	< 0.001	2.78(2.30,3.38)	< 0.001
Females	1	0.0	1.000	2.10(0.90,4.90)	0.086
**Year**					
After 2015	15	91.5	< 0.001	2.48(2.06,3.00)	< 0.001
Before 2015	16	98.0	< 0.001	3.06(2.24,4.18)	< 0.001
**Continent**					
Asia	14	90.7	< 0.001	2.50(2.03,3.09)	< 0.001
Europe	13	98.4	< 0.001	3.11(2.22,4.36)	< 0.001
North America	2	0.0	0.813	2.77(2.43,3.17)	< 0.001
Oceania	2	0.0	0.712	2.29(1.57, 3.35)	< 0.001
**Sampling method**					
Simple random	7	96.2	0.001	2.47(1.63,3.76)	< 0.001
Cluster random	17	93.4	< 0.001	2.67(2.23,3.21)	< 0.001
General survey	4	82.1	< 0.001	3.80(2.50,5.77)	< 0.001
**Types of bullying**					
Traditional	16	98.0	< 0.001	2.38(1.89,3.00)	< 0.001
Cyber	11	79.5	< 0.001	2.93(2.26, 3.78)	< 0.001
Traditional and cyber	4	79.0	0.003	4.49(3.30,6.12)	< 0.001
**Article quality**					
Medium quality	21	96.8	< 0.001	2.72( 2.17,3.42)	< 0.001
High quality	10	91.3	< 0.001	2.85(2.32,3.50)	< 0.001

## Discussion

A total of 31 cross-sectional studies on the relationship between bullying and children and adolescents were included in this study. The results showed that bullying was a risk factor for depression in children and adolescents [OR = 2.77, 95% CI (2.29, 3.35), *P* < 0.001] (Fig. 2). Additionally, 11 studies showed that bullying children and adolescents also had a risk of depression [OR = 1.73, 95% CI (1.34, 2.23), *P* < 0.001] (Fig. 4). Furthermore, this study found that bullying—bullying children and adolescents have a higher risk of depression than the former two [OR = 3.19, 95% CI (2.54, 4.01), *P* = 0.001] (Fig. 5).

No previous meta-analysis has explicitly focused on the relationship between depression in children and adolescents and bullying, being bullied, bullying—being bullied. Our study is the first meta-analysis that includes 31 cross-sectional surveys focusing on the relationship between bullying behavior and depression in children and adolescents. Lutrick K [51] reported a positive relationship between being bullied and depression in children and adolescents through a meta-analysis, consistent with the results of the current study; however, their meta-analysis only evaluated the relationship between being bullied and depression in children and adolescents and focused on Latino populations, which are understudied. Recently, a meta-analysis conducted by Moore SE [52] found that bullying has negative impacts on mental health in children and adolescents, but the type of bullying examined was limited, and no systematic review of other types of bullying behavior has been conducted. Gini G demonstrated [53] an association between bullying and psychosomatic problems through a meta-analysis. However, the outcome of this meta-analysis was the incidence of psychosomatic problems in children and adolescents; although these psychosomatic problems included depression, the research object was not specific. In contrast, the target of this meta-analysis is depression. The target population is more accurate, covering a wider range of people (including people from Europe and Asia) and a larger sample size. We also evaluated the effects of bullying and bullying-bullied behavior on depression. The results may inform the prevention and control of depression in children and adolescents.

This meta-analysis showed that bullying is related to depression in children and adolescents, which is consistent with the findings of Fan H and Gao L [54, 55]. This association may be because adolescents who experience bullying perceive themselves more negatively, are more closed off, and are reluctant to seek outside support. A study by Duan S et al. showed that when children and adolescents are bullied, their emotions are greatly affected; this emotional harm is difficult to treat and reduces the individual's mental health, resulting in depression [56]. Additionally, victims of bullying are regarded as weak, even though they may have excellent grades in school. After being bullied, the victims are typically threatened with harm if they tell an adult [57]. These findings suggest that children and adolescents are at risk of suffering from depression due to bullying. While controlling bullying, the mental health problems of children and adolescents after bullying are still one of the key tasks of our medical care.

This meta-analysis found that bullying is a risk factor for depression in children and adolescents. This finding is consistent with that of Choi JK [55]. One explanation may be that bullying individuals have depression and choose to use aggression as a coping mechanism. In addition, bullying individuals are unable to normally communicate with their peers; thus, they are rejected and experience depression(Lee 2021). Relevant studies have shown that aggressive bullying individuals are prone to depressive symptoms and even self-loathing thoughts and behaviors, which may be related to symptoms that have a high co-occurrence with aggression, such as impulsivity and anger [56]. Therefore, we should not only guide the psychology of children and adolescents correctly to reduce the occurrence of bullying but also draw attention to the psychological health of children and adolescents who implement bullying in society, which is a problem that we cannot ignore.

The meta-analysis also found that of the three bullying categories, bullying-bullied behavior had the strongest association with depression in children and adolescents. A study in Macau, China showed that bullying-bullied individuals experienced the most negative emotions, such as depression and anxiety, and the lowest life satisfaction [57]. The probable cause is that bullying-bullied individuals experience the negative effects of both bullying and being bullied. These individuals exhibit poor psychosocial functioning, poor self-control, vulnerability to rejection from peer groups, and the highest levels of depression [58]. The meta-analysis results suggest that reducing bullying among children and adolescents will help to prevent and control the occurrence of depression. Additionally, psychological intervention may be needed for children and adolescents after experiences of bullying and being bullied.

The number of victims of cyberbullying has increased over the past decade, accompanied by increasing concern about the harmful effects of cyberbullying on victims. Multiple studies have linked traditional bullying among teenagers with depression, suicidal ideation, and nonfatal suicidal behavior [59, 60]. However, the psychological outcomes of cyberbullying are inconsistent and unclear, possibly because of its recent development. Some authors have argued that the consequences of cyberbullying are similar to those of traditional bullying [37, 61]; others believe that cyberbullying is more distressing than traditional bullying [62].

The subgroup analysis in this study showed that the risk of depression after being bullied in children and adolescents was significantly higher after 2015 than that before 2015. One explanation is that the recent technological advances and the internet age have facilitated the appearance of cyberbullying in the lives of children and adolescents. Thus, some children and adolescents may not only experience traditional bullying but also cyberbullying. As mentioned earlier, adolescence is a critical time for psychological development; thus, adolescents are at higher risk of depression. The results of this meta-analysis also indicate that the risk of depression in children and adolescents after being bullied is higher in Europe than in Asia; this may be because European countries carry out universal screening for depression in adolescents. In addition, in terms of screening tools, the Epidemic Investigation Center Depression Scale, the Children's Depression Scale, and the Patient Health Questionnaire are widely used to screen for depression in Chinese children and adolescents [63, 64]. However, the screening ability of these scale need to be further verified and revised, and their psychometric properties (such as sensitivity, specificity, and diagnostic accuracy) should be determined according to different regions and survey samples. For example: the internal consistency for the CES-D was α = 0.78–0.79, but the subjects answered for a long time, had a high emotional load, and were sensitive to the project content. The content of the CES-DC is similar to that of the CES-D, but the former is more applicable to children and adolescents aged 6 to 17 years old because it uses simpler expressions [65]. The retest reliability of each item of SDS scale is 0.730 ~ 1.000, and the Cronbach α coefficients range from 0.782 ~ 0.784, indicating that it can be used for screening depression in adults and adolescents [66].

This study also found that the risk of depression was similar in children and adolescents who experienced traditional bullying or cyberbullying, suggesting that, while cyberbullying merits attention, school bullying should still be addressed. A variety of support should be included (e.g., from schools, relevant departments, and families) to bolster children’s mental health and provide timely support. Additionally, as traditional bullying, cyberbullying, and other types of bullying are related, more thorough and comprehensive bullying prevention programs and regulations are warranted to effectively reduce bullying among children and adolescents to promote healthy development.

In this meta-analysis, the heterogeneity of the included studies was high; after subgroup analysis (according to sex, sampling method, publication year, and region), *I*^*2*^ was still greater than 50%, suggesting that these factors may not have been the source of heterogeneity. Since the 31 included studies were from 13 countries, the definition or assessment of bullying and depression, location, participant ethnicity, and culture may have all contributed to the heterogeneity.

### Limitations

Only cross-sectional data were included in this meta-analysis, which precludes determination of causal associations. Additionally, few studies have been conducted on bullying, bullying-bullied behavior and depression in children and adolescents, which may reduce the reliability of the results. Subgroup analysis was carried out to analyze the heterogeneity among studies. The results of the funnel plot and Egger's test showed that publication bias was present, suggesting that it may be caused by publication type (i.e., gray literature).

## Conclusion

This meta-analysis shows that there is a significant correlation between depression and bullying, being bullied, and bullying-bullied behavior among children and adolescents. All three experiences are risk factors for depression. Subgroup analysis revealed that children and adolescents who have been bullied—bullied are most likely to suffer from depression. Ideally, these findings will inform and guide the development of preventive measures, thereby promoting the physical and mental health development of children and adolescents.

## Data Availability

All data analyzed during this study are included in this published article and the original studies’ publications.
